# The role of triglycerides in predicting new-onset arthritis in the general population over 45 years old: evidence from the China health and retirement longitudinal study

**DOI:** 10.3389/fendo.2025.1530874

**Published:** 2025-05-08

**Authors:** Weiwei Ma, Honggu Chen, Jing Deng, Qipeng Yuan, Huanan Li

**Affiliations:** ^1^ Clinical Medical College, Jiangxi University of Chinese Medicine, Nanchang, China; ^2^ Orthopaedics Department, Taizhou Hospital of Zhejiang Province, Taizhou, China; ^3^ Graduate School, Guangxi University of Chinese Medicine, Nanning, China; ^4^ Orthopaedics Department, Hospital Affiliated to Jiangxi University of Chinese Medicine, Nanchang, China

**Keywords:** arthritis, triglycerides, lipids, China health and retirement longitudinal study, longitudinal evidence

## Abstract

**Background:**

Arthritis is a common degenerative joint disease with a high prevalence especially in the elderly population. Due to its strong association with chronic pain and dysfunction, arthritis has become an important challenge in public health. Recent studies have shown that triglyceride (TG) levels, as key metabolic markers, may play an important role in the pathogenesis of arthritis, and its associated inflammatory response may accelerate joint degeneration and inflammatory process.

**Objective:**

Based on the above findings, the aim of this study was to investigate the association between baseline TG levels and the incidence of arthritis in adults aged 45 years and older, utilizing data from the China Health and Retirement Longitudinal Study(CHARLS).

**Methods:**

This study utilized the CHARLS from 2011 to 2018, which included 7,551 participants aged 45 years and older. The association between TG levels and new-onset arthritis was assessed by logistic regression modeling, adjusting for demographic and health-related variables. The potential role of HDL-C, LDL-C, and BMI in the TG-arthritis association was further assessed by mediation analysis, which decomposed the association into direct and indirect effects.

**Results:**

During the study period, 3,363 participants (44.5%) developed arthritis. Higher TG levels were significantly associated with arthritis risk, with an 8% increase in arthritis risk for each interquartile range (IQR) increase in TG (OR=1.08; 95% CI, 1.039-1.137.) Interquartile analyses of TG levels showed a significant dose-response trend (*P* trend <0.05), suggesting that the risk of arthritis tended to rise progressively with higher TG levels. Mediation analysis further revealed that HDL-C mediated approximately 43.5% of the TG-arthritis association, suggesting an important role of HDL-C in the metabolic pathway of arthritis development.

**Conclusion:**

Elevated TG levels were significantly associated with an increased risk of arthritis, and this association was partially mediated by HDL-C. The findings suggest that interventions targeting reduced TG levels and enhanced HDL function may have potential value in arthritis prevention. Future studies should focus on lipid metabolism intervention strategies to reduce arthritis risk and delay disease progression, providing a new scientific basis for arthritis management.

## 1 Introduction

Arthritis, a degenerative inflammatory disorder associated with aging, is primarily categorized into two major subtypes: osteoarthritis (OA) and rheumatoid arthritis (RA). Both conditions are characterized by progressive cartilage degradation and subchondral bone deterioration (1). With the global trend of population aging, the prevalence of arthritis is expected to rise continuously, posing an increasingly severe public health challenge. This disease not only leads to joint dysfunction and chronic pain but also significantly compromises patients’ quality of life. Moreover, arthritis exerts a substantial economic burden on families and society due to its negative impact on work capacity and household income (2, 3). Although the precise pathogenesis of arthritis remains incompletely elucidated, current clinical treatment strategies primarily focus on symptom management rather than achieving a cure. Consequently, many patients ultimately require joint replacement surgery, imposing a significant burden on both individuals and healthcare systems. Despite increasing medical expenditures, numerous patients continue to suffer from persistent pain, functional limitations, and psychological distress, with only marginal improvements in overall quality of life. This situation underscores the urgent need for more effective therapeutic and intervention strategies in clinical practice. Notably, the integration of early diagnosis with targeted treatment strategies holds great promise for improving comprehensive health management in arthritis patients. Such an approach may contribute to mitigating the long-term social and economic burdens associated with the disease.

Various factors, including environmental exposure and overall health status, contribute to the development of arthritis. Key risk factors include age, sex, racial differences, inflammatory responses, and metabolic disorders (4, 5). In recent years, with the emergence of the concept of metabolic arthritis, accumulating research evidence has highlighted the critical role of metabolic imbalance in the onset and progression of arthritis (6, 7). This insight has spurred further investigations into the association between metabolic biomarkers—such as blood lipid and glucose levels—and arthritis. Among these biomarkers, triglyceride (TG) levels, as a fundamental indicator of metabolic health, have garnered significant attention due to their potential involvement in arthritis pathogenesis. Studies have demonstrated that elevated TG levels in obese individuals are significantly correlated with an increased risk of arthritis, potentially attributable to the chronic low-grade inflammatory state associated with obesity (8). Moreover, some studies suggest that high TG levels may directly contribute to the pathological mechanisms of joint inflammation, offering new perspectives for understanding arthritis pathogenesis (9). Based on these findings, TG level regulation has been proposed as a potential strategy for arthritis prevention and treatment. To further elucidate the relationship between TG levels and arthritis among middle-aged and elderly populations in China, this study utilizes data from the China Health and Retirement Longitudinal Study (CHARLS). The objective is to systematically evaluate the causal relationship between TG levels and arthritis risk, thereby providing scientific evidence to support the early prevention and precise intervention of metabolic arthritis.

## 2 Methods

### 2.1 Study population

CHARLS is a nationally representative longitudinal cohort study focusing on individuals aged 45 and above in China, designed to provide comprehensive and long-term data on the health and socioeconomic status of middle-aged and elderly populations. Initiated in 2011, the study employs a multistage probability proportional-to-size sampling method, selecting approximately 10,000 households from 150 counties across 28 provinces, with over 17,000 individuals enrolled. The survey encompasses a broad spectrum of areas, including demographic characteristics, health status, medical insurance, intra-household economic transfers, employment, income, expenditures, and assets. Data collection is conducted through face-to-face interviews utilizing computer-assisted personal interview (CAPI) technology by rigorously trained interviewers, ensuring data accuracy and reliability. To maintain data continuity and completeness, CHARLS follows up with participants every 2–3 years. Notably, in the 2011 and 2018 surveys, participants who had fasted for at least 12 hours provided fasting venous blood samples. All biological samples were collected by certified professionals following standardized operating procedures and were promptly transported at 4°C to the Clinical Laboratory of Youan Hospital, Capital Medical University, in Beijing. The laboratory employed enzymatic colorimetric methods to measure key metabolic indicators, including triglycerides (TG), low-density lipoprotein cholesterol (LDL-C), and high-density lipoprotein cholesterol (HDL-C). Additionally, the detailed CHARLS questionnaire and methodology have been comprehensively documented in previous studies (10).

**Figure 1:**
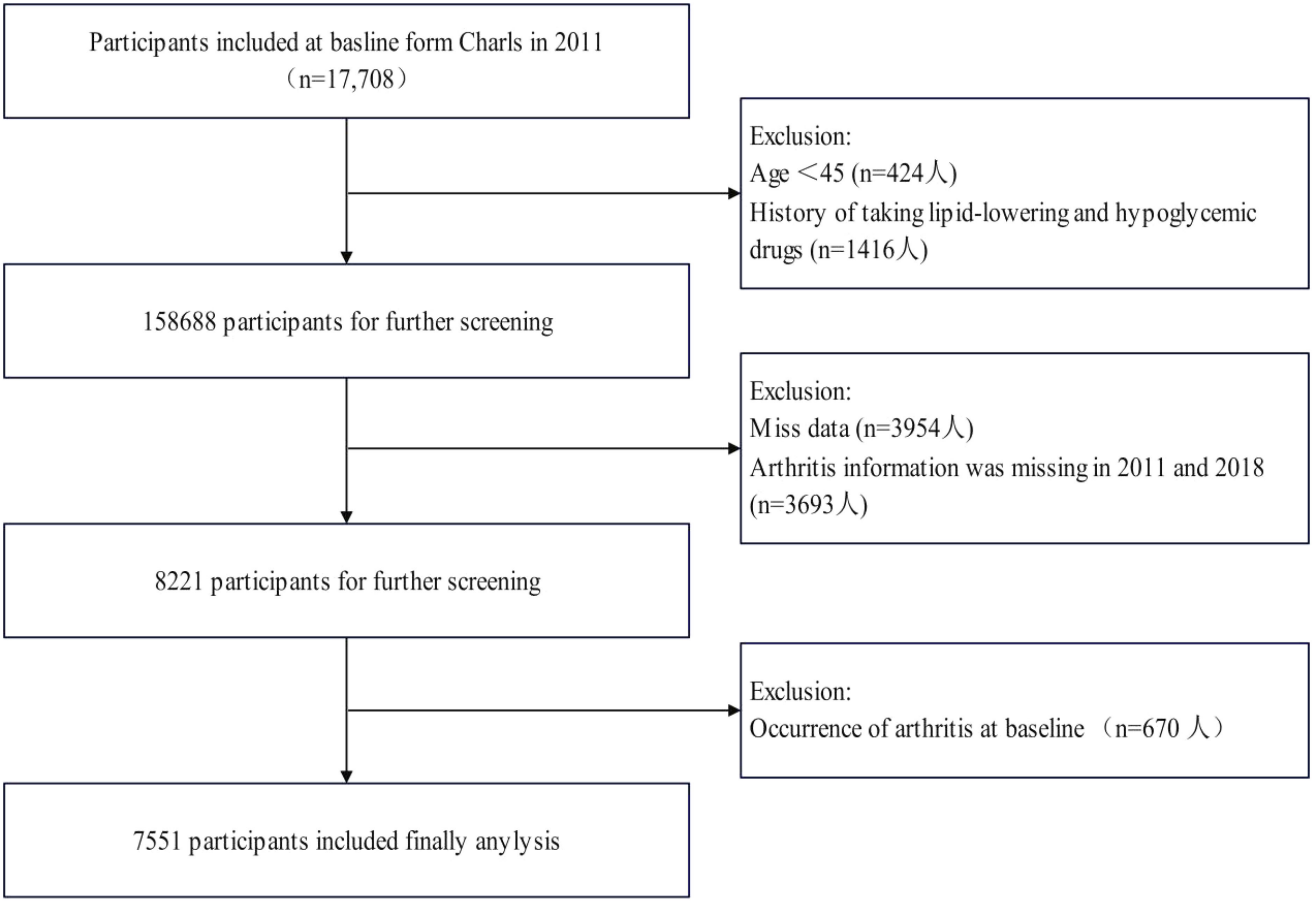
Flow chart for the selection of participants in the cohort study from Charls from 2011 to 2018 (n=7551).

**Figure 1** systematically delineates the participant selection flowchart within the study cohort. From the 17,708 individuals who completed baseline assessments (including physical examinations and questionnaire surveys) in 2011, sequential exclusions were implemented based on predefined criteria: 424 participants aged <45 years, 1,416 subjects with histories of lipid-lowering or glucose-modifying pharmacotherapy, 3,954 cases with incomplete datasets, 3,693 individuals lacking arthritis diagnostic records in both 2011 and 2018 follow-ups, and 670 baseline arthritis diagnoses. Medication histories were ascertained through self-reported responses to the CHARLS standardized questionnaire item: “Are you currently undergoing any therapeutic interventions for [dyslipidemia/diabetes/hyperglycemia] or related complications? (Select all applicable options)”, with response categories encompassing Western pharmaceuticals, traditional Chinese medications, and alternative therapies.

### 2.2 Assessment of arthritis events

In this study, “incident arthritis” is defined as cases of arthritis first diagnosed by a physician during the follow-up period from 2011 to 2018. Self-reported arthritis diagnosed by physicians is commonly utilized in epidemiological studies, with data regarding the outcome obtained through a questionnaire (11, 12). Participants who answered affirmatively to the question “Have you been diagnosed with arthritis or rheumatism by the doctor?” (CHARLS) or “Has a doctor or other health professional ever told you that you had arthritis?”. To ensure the accuracy of the study population, individuals who had already reported an arthritis diagnosis in the 2011 baseline survey were excluded. According to this definition of incident arthritis, only participants who were first diagnosed with arthritis during the 2011–2018 follow-up period were included in the study.

### 2.3 Assessment of covariates

The covariates included in this study encompass sociodemographic characteristics, lifestyle behaviors, and current health status. Sociodemographic characteristics include age (years), sex (male/female), marital status, education level (below primary school/primary school/junior high school/senior high school), sleep duration, and residence (rural/urban).Lifestyle behaviors were assessed based on smoking status (Former smoking status/Current smoking status) and drinking status (Past alcohol consumption/Present alcohol consumption). Current health status includes the diagnosis of hypertension and diabetes. Hypertension was defined based on clinical blood pressure measurements (systolic blood pressure ≥140 mmHg or diastolic blood pressure ≥90 mmHg), self-reported hypertension diagnosis during follow-up, or the use of antihypertensive medication. Diabetes was diagnosed according to the 2005 American Diabetes Association criteria, including fasting blood glucose level ≥126 mg/dL (7 mmol/L), random blood glucose level ≥200 mg/dL (11.1 mmol/L), glycated hemoglobin (HbA1c) level ≥6.5%, or self-reported diabetes or hyperglycemia diagnosis. Additionally, laboratory indicators included triglycerides (TG), low-density lipoprotein cholesterol (LDL-C), high-density lipoprotein cholesterol (HDL-C), and body mass index (BMI). BMI was categorized according to Chinese adult standards: underweight (BMI <18.5), normal weight (18.5 ≤ BMI <24), overweight (24 ≤ BMI <28), and obesity (BMI ≥28).

### 2.4 Statistical analysis

The data utilized in this study were obtained from the China Health and Retirement Longitudinal Study conducted between 2011 and 2018, encompassing a total of 7,551 participants. Statistical analyses were performed using a range of appropriate methods (13). For continuous variables with normal distribution, results were expressed as means and standard deviations (SDs), while categorical variables were summarized using frequencies and percentages. Differences in baseline characteristics among groups were assessed using the χ² test or analysis of variance (ANOVA), as appropriate.

To investigate the association between triglyceride (TG) levels and incident arthritis, multivariable logistic regression was employed to estimate adjusted odds ratios (ORs) and 95% confidence intervals (CIs) for cognitive impairment, whereas multivariable linear regression was used to evaluate continuous cognitive performance measures. Three hierarchical models were developed: Model 1 (unadjusted); Model 2 (adjusted for demographic factors including age, sex, education level, residence, marital status, and body mass index); and Model 3 (fully adjusted for all covariates). Stratified subgroup analyses were conducted to assess potential effect modification across predefined demographic and clinical subgroups. In addition, interaction analyses were performed to explore whether sociodemographic and health-related factors modulate the association between TG levels and arthritis risk, thereby providing further insight into the underlying mechanisms. The dose-response relationship between TG levels and the risk of new-onset arthritis was examined using restricted cubic spline (RCS) functions with four knots positioned at the 5th, 35th, 65th, and 95th percentiles. Finally, Mediation analysis was conducted using the “mediation” package in R (14), which decomposes the effects into direct and indirect components to quantify the impact of exposure on outcomes and to elucidate the causal structure between variables.

All statistical analyses were conducted using R software (version 4.4.2). RCS analysis was implemented via the “ANOVA” function, and mediation analysis was performed using the “mediation” package. A two-sided P-value of <0.05 was considered statistically significant.

## 3 Results

### 3.1 Basic characteristics of study participants

A total of 7,551 participants were included in this study, with an average age of 57 years, comprising 3,307 males and 4,244 females. During the 9-year follow-up period from 2011 to 2020, a total of 3,363 participants were diagnosed with arthritis.

**Table 1** presents the baseline characteristics of participants stratified by arthritis status. Participants with arthritis were generally older, with a significantly higher median age and BMI, as well as shorter sleep duration (*P*<0.001). The incidence of arthritis was notably higher in women than in men (*P*<0.001). Additionally, the proportion of individuals living alone was higher in the arthritis group compared to the non-arthritis group (*P*=0.03). Individuals with arthritis were also more likely to have lower education levels, reside in rural areas, and report smoking or drinking habits. However, no significant differences were observed between the arthritis and non-arthritis groups in terms of hypertension (*P*=0.893) and diabetes (*P*=0.701) prevalence. These findings underscore specific demographic and lifestyle factors associated with arthritis, offering valuable insights for risk assessment and the development of targeted prevention strategies.

**Table 1:** Baseline characteristics of the study population by arthritis status at follow-up.

Variable	Total (N= 7551)	New-onset arthritis?		*P* value
		No (N = 4188)	Yes(N = 3363)	
Age, Median (Q1,Q3)	57 (51, 63)	57 (50, 63)	58 (52, 64)	< 0.001
Sex, N (%)				< 0.001
Female	4244 (56.2)	2168 (51.77)	2076 (61.73)	
Male	3307 (43.8)	2020 (48.23)	1287 (38.27)	
Marry, N (%)				0.03
Non-Married	740 (9.8)	382 (9.12)	358 (10.65)	
Married	6811 (90.2)	3806 (90.88)	3005 (89.35)	
Education, N (%)				< 0.001
Below primary school	3537 (46.84)	1767 (42.19)	1770 (52.63)	
Primary school	1674 (22.17)	930 (22.21)	744 (22.12)	
Middle school	1592 (21.08)	989 (23.62)	603 (17.93)	
High school and above	748 (9.91)	502 (11.99)	246 (7.31)	
Location, N(%)				< 0.001
City/Town	1152 (15.26)	735 (17.55)	417 (12.4)	
Village	6399 (84.74)	3453 (82.45)	2946 (87.6)	
Past alcohol consumption				< 0.001
No	4716 (62.46)	2540 (60.65)	2176 (64.7)	
Yes	2835 (37.54)	1648 (39.35)	1187 (35.3)	
Present alcohol consumption				< 0.001
No	5123 (67.85)	2732 (65.23)	2391 (71.1)	
Yes	2428 (32.15)	1456 (34.77)	972 (28.9)	
Former smoking status				< 0.001
No	4785 (63.37)	2557 (61.06)	2228 (66.25)	
Yes	2766 (36.63)	1631 (38.94)	1135 (33.75)	
Current smoking status				< 0.001
No	5357 (70.94)	2877 (68.7)	2480 (73.74)	
Yes	2194 (29.06)	1311 (31.3)	883 (26.26)	
Hibpe, N (%)				0.893
No	4117 (54.52)	2280 (54.44)	1837 (54.62)	
Yes	3434 (45.48)	1908 (45.56)	1526 (45.38)	
Diabe, N(%)				0.701
No	6529 (86.47)	3615 (86.32)	2914 (86.65)	
Yes	1022 (13.53)	573 (13.68)	449 (13.35)	
Sleep, Median (Q1,Q3)	6.5 (5, 8)	7 (6, 8)	6 (5, 8)	< 0.001
BMI, Median (Q1,Q3)	23.27 (21.03, 25.91)	23.2 (20.97, 25.7)	23.36 (21.13,26.14)	0.008
HDL-C (mg/dl), Median (Q1,Q3)	49.48 (40.59, 59.92)	49.1 (40.11,59.63)	50.26 (40.98, 59.92)	0.014
LDL-C (mg/dl), Median (Q1,Q3)	114.82 (93.75, 137.24)	114.43 (93.56,136.47)	115.21 (93.94,138.4)	0.188

BMI, body mass index; HDL-C, high-density lipoprotein cholesterol; LDL-C, low-density lipoprotein cholesterol.

### 3.2 Baseline characteristics of study participants

**Table 2** summarizes the baseline characteristics of study participants stratified into quartiles based on TG levels. As TG levels increased, the prevalence of hypertension and diabetes also showed a significant rise, with the highest TG quartile exhibiting hypertension and diabetes prevalence rates of 55.33% and 23.25%, respectively, demonstrating a significant statistical trend (*P*<0.001).Furthermore, other baseline characteristics also varied significantly with TG levels, including sex and age distribution, smoking and drinking habits, and place of residence (all *P*<0.05). These findings highlight the association between TG levels and various health and demographic factors, suggesting that TG management may play a crucial role in controlling related health risks.

**Table 2:** Baseline characteristics of the Study Population According to Triglyceride Index Quartiles.

Variable	Total (N = 7551)	Q1 (N= 1940)	Q2 (N = 1869)	Q3 (N = 1875)	Q4 (N = 1867)	*P* value
Age, Median (Q1,Q3)						*P*< 0.001
Sex, N (%)	4244 (56.2)	929 (47.89)	1037 (55.48)	1155 (61.6)	1123 (60.15)	
Female	3307 (43.8)	1011 (52.11)	832 (44.52)	720 (38.4)	744 (39.85)	
Male						0.284
Marry, N (%)	740 (9.8)	210 (10.82)	183 (9.79)	179 (9.55)	168 (9)	
Non-Married	6811 (90.2)	1730 (89.18)	1686 (90.21)	1696 (90.45)	1699 (91)	
Married	58.00 (52.00, 64.00)	60.00 (54.00, 66.00)	59.00 (53.00, 64.00)	57.50 (51.75, 63.00)	58.00 (51.00, 64.00)	0.041
Education, N (%)						0.161
Below primary school	3537 (46.84)	917 (47.27)	861 (46.07)	918 (48.96)	841 (45.05)	
Primary school	1674 (22.17)	417 (21.49)	444 (23.76)	407 (21.71)	406 (21.75)	
Middle school	1592 (21.08)	424 (21.86)	377 (20.17)	368 (19.63)	423 (22.66)	
High school and above	748 (9.91)	182 (9.38)	187 (10.01)	182 (9.71)	197 (10.55)	
Location, N(%)						*P*< 0.001
City/Town	1152 (15.26)	255 (13.14)	270 (14.45)	285 (15.2)	342 (18.32)	
Village	6399 (84.74)	1685 (86.86)	1599 (85.55)	1590 (84.8)	1525 (81.68)	
Past alcohol consumption						*P*< 0.001
No	4716 (62.46)	1114 (57.42)	1173 (62.76)	1236 (65.92)	1193 (63.9)	
Yes	2835 (37.54)	826 (42.58)	696 (37.24)	639 (34.08)	674 (36.1)	
Present alcohol consumption						*P*< 0.001
No	5123 (67.85)	1202 (61.96)	1280 (68.49)	1347 (71.84)	1294 (69.31)	
Yes	2428 (32.15)	738 (38.04)	589 (31.51)	528 (28.16)	573 (30.69)	
Former smoking status						*P*< 0.001
No	4785 (63.37)	1128 (58.14)	1168 (62.49)	1275 (68)	1214 (65.02)	
Yes	2766 (36.63)	812 (41.86)	701 (37.51)	600 (32)	653 (34.98)	
Current smoking status						*P*< 0.001
No	5357 (70.94)	1284 (66.19)	1294 (69.23)	1408 (75.09)	1371 (73.43)	
Yes	2194 (29.06)	656 (33.81)	575 (30.77)	467 (24.91)	496 (26.57)	
Hibpe, N (%)						*P*< 0.001
No	4117 (54.52)	1246 (64.23)	1079 (57.73)	958 (51.09)	834 (44.67)	
Yes	3434 (45.48)	694 (35.77)	790 (42.27)	917 (48.91)	1033 (55.33)	
Diabe, N(%)						*P*< 0.001
No	6529 (86.47)	1776 (91.55)	1685 (90.16)	1635 (87.2)	1433 (76.75)	
Yes	1022 (13.53)	164 (8.45)	184 (9.84)	240 (12.8)	434 (23.25)	
Sleep, Median (Q1,Q3)	6.5 (5, 8)	7 (5, 8)	6 (5, 8)	6 (5, 8)	7 (5, 8)	0.684
BMI, Median (Q1,Q3)	23.27 (21.03, 25.91)	21.92 (20, 23.95)	22.88 (20.78, 25.26)	23.79 (21.27, 26.44)	24.87 (22.64, 27.47)	*P*< 0.001
HDL-C (mg/dl), Median (Q1,Q3)	49.48 (40.59, 59.92)	59.54 (50.64, 69.2)	52.58 (44.85, 61.47)	47.17 (40.59, 55.28)	39.05 (32.86, 46.39)	*P*< 0.001
LDL-C (mg/dl), Median (Q1,Q3)	114.82 (93.75, 137.24)	107.86 (90.08, 127.19)	117.14 (97.42, 138.4)	122.55 (101.29, 144.59)	112.89 (86.99, 139.18)	*P*< 0.001

Different statistical methods were used to calculate p-values based on variable types. Specifically, for categorical variables, the chi-square test was used. For continuous variables, the method depended on distribution: the rank-sum test was applied to non-normally distributed variables, while ANOVA was used for normally distributed variables. BMI, body mass index; HDL-C, high-density lipoprotein cholesterol; LDL-C, low-density lipoprotein cholesterol.

### 3.3 Analysis of the association between triglycerides and arthritis incidence

**Figure 2:**
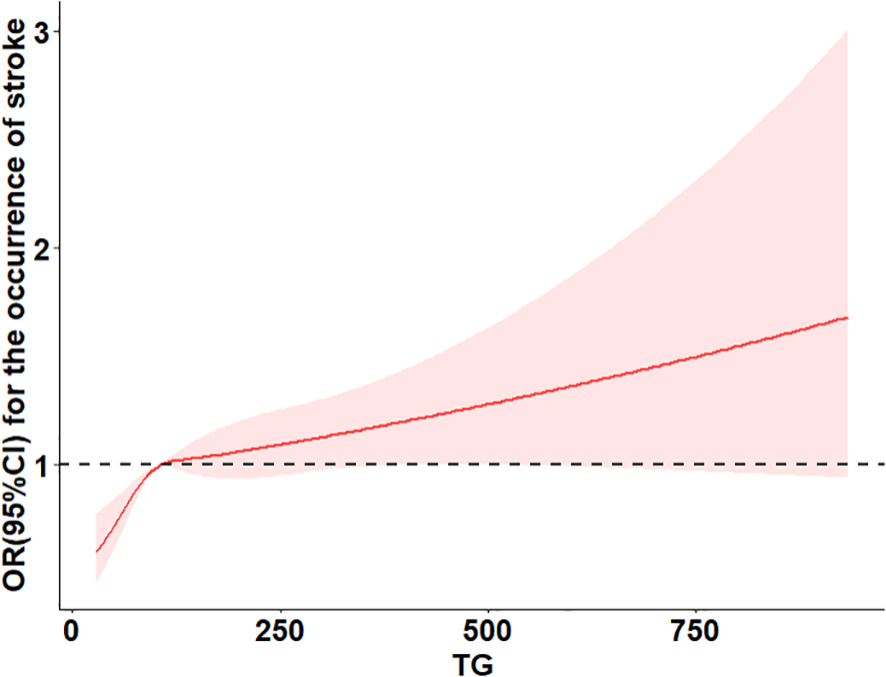
Restricted cubic spline curve for triglycerides and risk of arthritis.

**Table 3** summarizes the association between TG levels and the risk of arthritis. In Model 1, using the lowest TG quartile (Q1) as a reference, the odds ratios (ORs) for arthritis in Q2, Q3, and Q4 were 1.09 (95% CI 0.95–1.23, *P*=0.21), 1.28 (95% CI 1.13–1.45, *P*<0.001), and 1.16 (95% CI 1.02–1.31, *P*=0.027), respectively. The P-value for trend was significant (*P* for trend = 0.0036), indicating a dose-response relationship, suggesting that higher TG levels may increase arthritis risk. In Model 2, after further adjusting for covariates, the ORs for Q2, Q3, and Q4 were 1.00 (95% CI 1.00–1.00, *P*=0.004), 0.69 (95% CI 0.60–0.80, *P*<0.001), and 0.99 (95% CI 0.84–1.16, *P*=0.907), respectively, with a trend P-value of 0.0043, still indicating a significant trend across quartiles. In the fully adjusted Model 3, the ORs for Q2, Q3, and Q4 were 1.00 (95% CI 1.00–1.00, *P*<0.001), 0.71 (95% CI 0.61–0.82, *P*<0.001), and 1.00 (95% CI 0.85–1.17, *P*=0.966), with the trend P-value remaining significant (0.0003). When TG was analyzed as a continuous variable, each interquartile range (IQR) increase was associated with an 8% higher risk of arthritis (OR=1.08; 95% CI 1.039–1.137). However, in the fully adjusted model, no significant dose-response relationship was observed between TG levels and arthritis risk (*P* overall = 0.067, *P* non-linear = 0.212).These findings suggest a potential association between TG levels and arthritis risk, but further analyses are required to confirm possible non-linear relationships. The results are illustrated in **Figure 2**.

**Table 3:** Logistic regression analysis of estimated triglycerides and arthritis.

-	Model 1	P	Model 2	P	Model 3	P
TG Per IQR	1.04(1.,1.09)	<0.001	1.05(1.01,1.10)	<0.001	1.08(1.039,1.137)	<0.001
Q1	ref		ref		ref	
Q2	1.09 (0.95, 1.23)	0.21	1.02 (0.97, 1.08)	0.004	1.06 (0.93, 1.21)	<0.001
Q3	1.28 (1.13, 1.45)	<0.001	1.19 (1.05, 1.32)	<0.001	1.22 (1.07, 1.39)	<0.001
Q4	1.16 (1.02, 1.31)	0.027	1.12 (0.98, 1.29)	0.907	1.12 (0.99, 1.28)	0.966
P for trend	0.0036		0.0043		0.0003	

Model 1 is the crude model without adjustments; Model 2 adjusts for age, sex, education level, residence, marital status, and body mass index; Model 3 further includes smoking status, alcohol consumption, sleep duration, diabetes, hypertension, low-density lipoprotein, and high-density lipoprotein.

### 3.4 Stratified analysis

**Figure 3:**
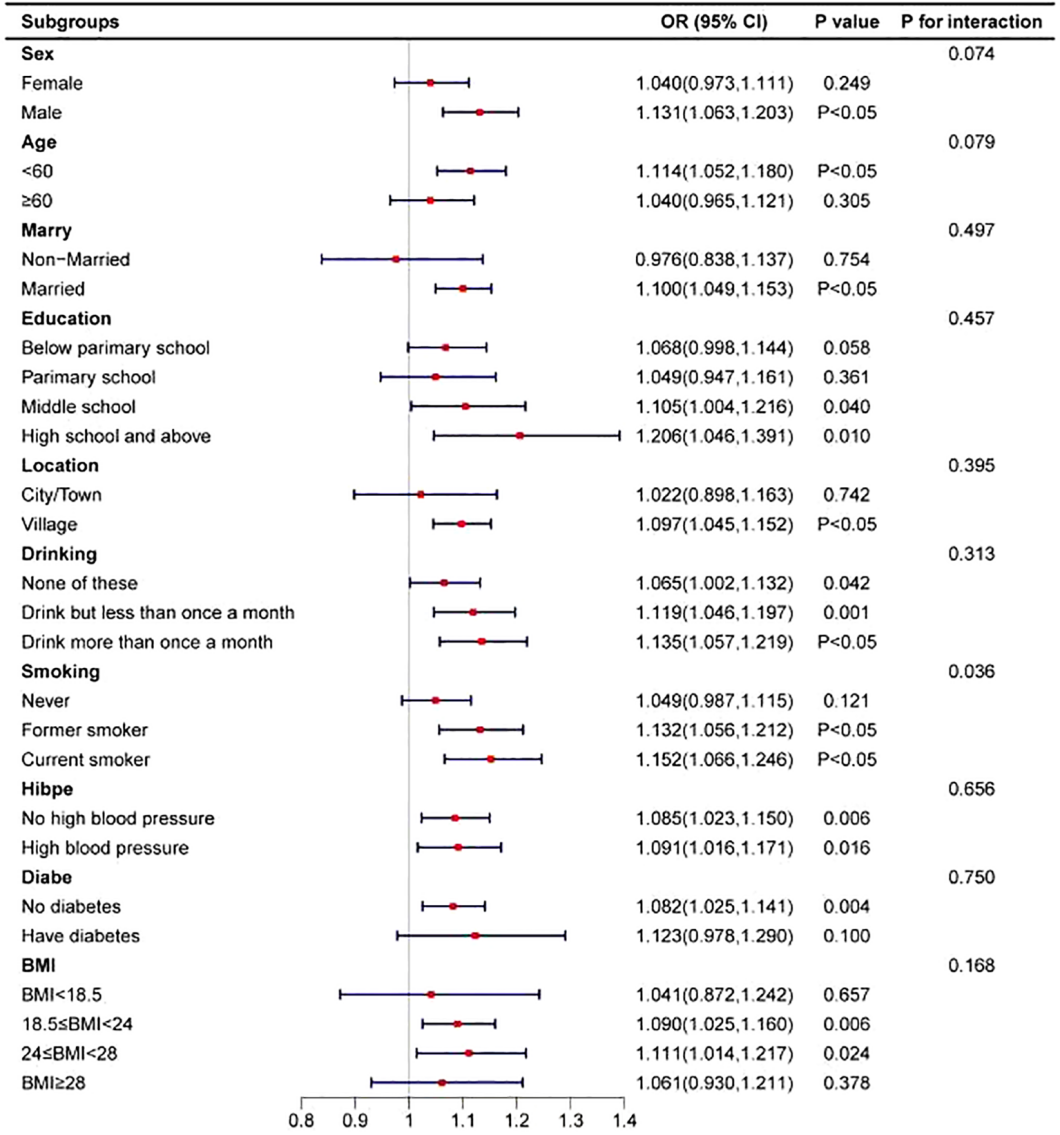
Association of triglycerides with arthritis in different subgroups.

To assess the stability of the positive correlation between TG levels and the risk of arthritis, participants were stratified based on sociodemographic factors and disease history, and the association was analyzed within each subgroup (see **Figure 3**). The results indicated a significant interaction between TG levels and smoking status (interaction *P*-value = 0.036), suggesting that smoking may modulate the relationship between TG levels and arthritis risk. Specifically, among current smokers, higher TG levels were associated with a significantly increased risk of arthritis. This finding highlights the potential impact of smoking on the association between TG levels and arthritis, suggesting that smokers with high TG levels may be at a higher risk of developing arthritis.

### 3.5 Mediation analysis

Mediation analysis revealed that the relationship between TG levels and arthritis was partially mediated by HDL. The average causal mediation effect (ACME) of HDL was -0.0000754 (95% CI: -0.000146 to -0.0000048; *P* < 2e-16), indicating a significant mediation effect. The average direct effect (ADE) was 0.000246 (95% CI: 0.000113 to 0.000379; *P* < 2e-16), suggesting that the effect of TG levels on arthritis was partially independent of HDL.A total of 43.5% of the overall effect of TG on arthritis was mediated by HDL (*P* < 2e-16), underscoring the critical role of HDL in this relationship. In contrast, LDL and BMI did not show significant mediation effects on the TG-arthritis association, suggesting that these factors may not be the primary mediators. The results are presented in **Table 4**.

**Table 4:** Intermediary analysis.

-	Estimate 95% CI	Lower 95% CI	Upper 95% CI	*P* value
TG-(HDL-C)-arthritis				
ACME (average)	−0.00007	−0.0001	−0.000004	<2e-16
ADE (average)	0.0002	0.0001	0.0003	<2e-16
Prop. Mediated (average)	-0.435	−3.22	-0.07	<2e-16
TG-(LDL-C)-arthritis				
ACME (average)	-0.0000012	-0.000017	0.000015	0.84
ADE (average)	0.0002	0.0001	0.0004	<2e-16
Prop. Mediated (average)	−0.0064	−0.0955	0.08	0.84
TG-BMI-arthritis				
ACME (average)	0.00000003	−0.000002	0.000002	1.00
ADE (average)	0.0003	0.0001	0.0004	<2e-16
Prop. Mediated (average)	0.000009	−0.009	0.01	1.00

CI, Confidence Interval; HDL-C, High-Density Lipoprotein Cholesterol; LDL-C, Low-Density Lipoprotein Cholesterol; ADE, Average Direct Effect; ACME, Average Causal Mediation Effect.

## 4 Discussion

This study systematically investigated the association between TG levels and the risk of arthritis among middle-aged and older adults in China, along with potential underlying mechanisms. Through comprehensive data analysis, a significant positive correlation was observed between serum TG levels and the incidence of arthritis. Notably, individuals with elevated TG levels had a markedly higher risk of developing arthritis compared to those with normal TG levels. This association remained robust after controlling for major confounding variables, including age, sex, educational attainment, residential location, marital status, smoking behavior, and alcohol consumption. Furthermore, HDL was identified as a key mediator in this relationship. These findings underscore the complex interplay between lipid metabolism and arthritis and offer valuable implications for the prevention and targeted management of arthritis.

Contemporary research on chronic diseases has increasingly shifted its focus from solely enhancing treatment efficacy to emphasizing primary prevention. This transition stems from the understanding that many chronic conditions, once established, are often irreversible and challenging to cure. In the context of arthritis prevention, identifying modifiable risk factors at the population level offers a critical “window of opportunity” to lower incidence rates and alleviate the societal burden of the disease (15, 16). Subgroup analyses in this study further substantiated the potential link between TG levels and arthritis risk. The results not only highlight the significance of monitoring TG levels but also provide compelling evidence supporting their role as predictive biomarkers for arthritis prevention, thereby broadening their relevance in public health interventions. Elevated TG levels may increase arthritis risk through various biological pathways. For example, a prospective cohort study reported a positive association between serum TG levels and the incidence of hand arthritis, suggesting that TG may serve as an independent risk factor (17). Similarly, a case-control study revealed a correlation between dyslipidemia and hand arthritis, further implicating TG in the pathogenesis of arthritis (18). These findings are consistent with the results of the present study, reinforcing the conclusion that TG is an independent risk factor for arthritis.

With the advancement of modern medical research, the understanding of the pathogenesis of arthritis has evolved from the simple mechanical wear theory to the systemic low-grade inflammation theory mediated by metabolic syndrome (19, 20). As one of the core components of metabolic syndrome (MetS), TG are not only significantly associated with cardiovascular metabolic risk factors such as central obesity, insulin resistance, HDL-C, and hypertension (21, 22), but also participate in the process of joint degeneration through multidimensional mechanisms. From a pathophysiological perspective, the pathogenic mechanisms of TG can be summarized as follows: first, at the biomechanical level, TG accumulation forms a positive feedback loop with obesity, accelerating cartilage matrix degradation by increasing joint load on the one hand, and on the other, promoting the abnormal secretion of pro-inflammatory cytokines such as IL-6 and TNF-α, as well as adipokines such as leptin and resistin, thereby creating an inflammatory microenvironment with both local and systemic interactions (23). Notably, the chronic low-grade inflammatory state inherent in metabolic syndrome can activate key signaling pathways such as NF-κB, significantly increasing the susceptibility of joint tissues to inflammatory damage (24). Second, at the level of immune regulation, TG metabolites influence both innate and adaptive immune responses through dual mechanisms: first, they activate the monocyte-macrophage system via the TLR4/MyD88 signaling axis, promoting M1 pro-inflammatory polarization, a mechanism validated in collagen-induced arthritis models—arthritis incidence and severity were significantly reduced in TLR4 knockout mice (25); second, they regulate the Th17/Treg cell balance, promoting the release of pro-inflammatory factors such as IL-17 and IL-23, with preclinical studies confirming that oleanolic acid acetate exerts joint protective effects by inhibiting Th1/Th17 cell expansion (26). Finally, TG-derived free fatty acids can induce mitochondrial dysfunction in synovial cells, leading to a rate of reactive oxygen species (ROS) production that exceeds the clearance capacity of antioxidant systems such as glutathione peroxidase, thereby triggering a lipid peroxidation chain reaction (27, 28).

Subgroup analysis results indicate that smoking plays a key moderating role in the relationship between triglycerides and arthritis incidence, a conclusion consistent with previous studies. Existing research has confirmed that smoking may increase the risk of arthritis, particularly among individuals positive for anti-citrullinated peptide antibodies (ACPA), where the risk is even more pronounced (29).Additionally, a study on U.S. adults found that current smokers had a significantly increased risk of developing arthritis, with a relative risk of 1.47 (95% CI 1.29–1.68) (30), suggesting that smoking may be an independent risk factor for arthritis. The impact of smoking on RA may be regulated by dosage and duration. Studies have shown that smoking is not only associated with the onset of RA but may also exacerbate disease progression by inducing oxidative stress. In a case-control study, the interaction between smoking and oxidative stress significantly increased the risk of arthritis, particularly in long-term smokers (31). This finding suggests that smoking may promote inflammation and contribute to arthritis development and progression by triggering oxidative stress. Beyond oxidative stress, smoking may also increase susceptibility to arthritis by altering immune system function. Research has shown that smoking can regulate gene expression in the immune system and serves as an important triggering factor for autoimmune diseases such as spondyloarthritis (SpA) and RA (32). Specifically, smoking can modulate cytokine levels, induce endothelial dysfunction, and alter epigenetic mechanisms, leading to greater disease severity in SpA patients who smoke. Further studies have elucidated the central role of smoking in the pathogenesis of arthritis, suggesting that smoking may exacerbate disease progression through activation of the aryl hydrocarbon receptor (AHR).AHR, as a transcription factor, regulates Th17 cell differentiation, which plays a crucial role in the development of autoimmune diseases. Experimental studies (33) have found that smoking can worsen arthritis and increase the proportion of Th17 cells, indicating that AHR activation may be an important mechanism by which smoking influences arthritis progression. Clinically, smoking not only affects the risk of RA onset but may also exacerbate disease severity and reduce treatment efficacy. Studies have shown that smokers exhibit higher disease activity and are more likely to become seropositive due to peptide citrullination. Moreover, these patients often show poorer treatment responses when receiving tumor necrosis factor (TNF) inhibitor therapy (32). These findings highlight the complex effects of smoking on the immune system and arthritis susceptibility, underscoring the need for further research to explore its underlying mechanisms and optimize arthritis prevention and treatment strategies.

Mediation analysis has revealed a key biological mechanism whereby HDL plays a significant mediating role in the relationship between TG levels and the incidence of arthritis. Studies have shown that HDL not only serves as a cholesterol transport carrier but also possesses multiple physiological functions, including antioxidant, anti-inflammatory, and cholesterol reverse transport-promoting properties, which may play an important role in the pathogenesis of arthritis (34). Previous research has confirmed that low HDL levels are associated with a higher incidence of various inflammatory diseases. For example, a study on polyarticular juvenile idiopathic arthritis found a higher prevalence of lipoprotein abnormalities among patients, particularly a reduction in HDL levels (35), suggesting that HDL may play a crucial role in the pathophysiological process of inflammatory joint diseases. Moreover, elevated TG levels may affect HDL function, particularly in the development of inflammatory diseases such as arthritis. Under normal conditions, HDL exerts anti-inflammatory effects, but its function may be impaired in an inflammatory state (36, 37). Research indicates that inflammatory responses can induce structural and functional changes in HDL, leading to a reduction in its antioxidant and anti-inflammatory capacities. These changes may be related to alterations in the phospholipid composition of HDL; for instance, an increase in lysophosphatidylcholine and a decrease in phosphatidylcholine content have been observed in patients with RA (38). Additionally, under chronic inflammatory conditions, the antioxidant enzyme activity of HDL, such as paraoxonase-1 (PON1), may be reduced, further weakening HDL’s anti-inflammatory and antioxidant functions (39, 40).

This study, based on the CHARLS database, explores the association between TG levels and arthritis, but several limitations remain. First, as an observational study, data from CHARLS cannot establish a causal relationship between TG levels and arthritis. Because the data are primarily derived from cross-sectional or longitudinal observations, the findings may be influenced by reverse causality or potential confounding factors, thus limiting the reliability of causal inferences. Second, the diagnosis of arthritis in CHARLS relies on self-reports from participants, which may lead to disease misclassification, particularly among individuals with mild or asymptomatic conditions. The reliance on self-reported data may compromise the accuracy and robustness of the study conclusions. In addition, the database lacks detailed information such as the frequency and duration of pain, making it difficult to distinguish between acute and chronic pain, which may differ in pathological mechanisms and exhibit distinct associations with metabolic health. Therefore, this limitation may affect the interpretability and clinical relevance of the findings to some extent. Lastly, CHARLS provides only a single time-point measurement of TG levels, making it difficult to capture their dynamic changes over time at the individual level. In contrast, longitudinal TG data would be more helpful in revealing the potential cumulative impact of TG levels on arthritis development, thereby providing a more comprehensive understanding of their association.

The results of this study indicate a significant association between elevated TG levels and an increased risk of arthritis, particularly among individuals aged 45 and older in China. This finding underscores the importance of monitoring TG levels and highlights the potential value of metabolic regulation as a preventive strategy for arthritis. Intervening in lipid metabolism may provide new avenues for delaying the onset and progression of arthritis. Future research could further explore the role of lipid-targeted interventions in arthritis management, offering new directions for delaying disease progression through metabolic health optimization.

## Data Availability

The original contributions presented in the study are included in the article/**Supplementary Material**. Further inquiries can be directed to the corresponding author/s.
